# The Effects of Oral Consumption of Honey on Key Metabolic Profiles in Adult Patients with Type 2 Diabetes Mellitus and Nondiabetic Individuals: A Systematic Review of Clinical Trials

**DOI:** 10.1155/2021/6666832

**Published:** 2021-01-23

**Authors:** Marzieh Akhbari, Masoumeh Jabbari, Mohammad Hossein Ayati, Nazli Namazi

**Affiliations:** ^1^School of Persian Medicine, Tehran University of Medical Sciences, Tehran, Iran; ^2^Student Research Committee, Department of Community Nutrition, Faculty of Nutrition Sciences and Food Industry, Shahid Beheshti University of Medical Sciences, Tehran, Iran; ^3^Endocrinology and Metabolism Research Center, Endocrinology and Metabolism Clinical Sciences Institute, Tehran University of Medical Sciences, Tehran, Iran; ^4^Department of History of Medicine, School of Traditional Medicine, Tehran University of Medical Sciences, Tehran, Iran; ^5^Diabetes Research Center, Endocrinology and Metabolism Clinical Sciences Institute, Tehran University of Medical Sciences, Tehran, Iran

## Abstract

**Objectives:**

Although several clinical trials have revealed the beneficial effects of honey on metabolic profiles, the results are conflicting. The aim of this study was to systematically summarize the effects of oral consumption of honey on key metabolic profiles in adult patients with type 2 diabetes mellitus (T2DM) and nondiabetic individuals.

**Methods:**

In total, four electronic databases, including PubMed/Medline, Web of Science, Scopus, and Cochrane library, were searched from 2000 to 31 July 2019 to identify all English language studies that would meet the eligibility criteria. Clinical trials which have examined the effects of oral consumption of any types of honey on anthropometric indices, glycemic status, lipid profiles, and blood pressure in both diabetic and nondiabetic adult subjects were included in the study.

**Results:**

Of the 7769 possible relevant studies (including 3547 duplicates) identified in the initial search, finally, 13 clinical trials were included in the systematic review. All studies except three had a parallel design. Of 13 studies, 8 trials did not have placebo/control groups. The included studies examined the impact of oral consumption of honey on glycemic status (*n* = 12), anthropometric indices (*n* = 6), lipid profiles (*n* = 10), and blood pressure (*n* = 3). Based on the Jadad scale, 5 studies had acceptable methodological quality, and the remaining (*n* = 8) had low methodological quality.

**Conclusion:**

The current systematic review showed that oral consumption of honey might have no significant effects on the modulation of metabolic profiles in nondiabetic subjects. In addition, a high intake of honey might increase glucose levels and worsen other metabolic parameters in patients with T2DM. Due to substantial heterogeneity in study design and limited clinical trials, results, however, should be interpreted with great caution.

## 1. Introduction

According to the report published by the World Health Organization (WHO), cardiovascular diseases (CVDs) are the leading cause of death worldwide, leading to about 17.9 million deaths per year [1]. Obesity, diabetes, and dyslipidemia play the pivotal roles in the incidence of CVDs [2, 3]. For the management of patients at high risk of CVDs, there are several strategies including taking medicines, lifestyle modifications, adherence to healthy diets [4], and consumption of functional foods [5, 6].

Functional foods can be considered as one of the useful modifiers of CVD risk factors [7]. Such types of food can either improve health status or reduce the risk of various diseases apart from providing nutritional requirements [8]. Leafy greens, berries, soy, fatty fish [9], and honey [10] are examples of functional foods.

Honey, a natural sweetener, is widely available across the world [11]. More than 300 different types of floral honey are available in the world's market places. The appearance, sensory characteristics, and the amount of biochemical components, including glucose to fructose ratio, mineral, and vitamin content of honey, vary based on botanical origin [12]. Honey is a high-carbohydrate food containing monosaccharide (glucose and fructose) and disaccharides [12, 13]. Therefore, its effects on glycemic parameters are exceedingly important, especially for patients with diabetes mellitus (DM) and those suffering from glucose intolerance. Due to differences in physicochemical properties of honey collected from various botanical sources, the glycemic index (GI) of honey varies between 32 and 85 [12].

Honey is beyond a carbohydrate source; it contains numerous components with antioxidant, anti-inflammatory, and antimicrobial characteristics including polyphenols, flavonoids, enzymes, vitamins, and trace elements [12, 14]. In traditional medicine such as traditional Persian medicine (TPM), honey has been used as a complementary therapy for wide ranges of diseases [15] such as liver and vascular diseases. In TPM, honey is called “Angabin” and “Shahd.” The type with no wax and transparent red color that is tasty and fragrance has been introduced as the best one [16, 17]. In the conventional medicine, honey has also been used for the treatment of gastrointestinal diseases, healing of ulcers (diabetic ulcers and bedsores), skin diseases, respiratory disorders, and urinary system diseases [15, 18, 19].

Several clinical trials have revealed the beneficial effects of honey on metabolic profiles, including lipid profiles, glycemic status, anthropometric indices, and inflammatory parameters [20–23]. However, the results are conflicting. To the best of our knowledge, no systematic review has been conducted to summarize the effects of oral consumption of honey on metabolic profiles. Accordingly, the primary objective of the present study was to examine the effects of honey on glycemic status, lipid profiles, anthropometric indices, and blood pressure in adult individuals, and the secondary objective was to compare its impacts on patients with type 2 diabetes (T2DM) and nondiabetic individuals and present findings quantitatively, if possible.

## 2. Methods

The current systematic review was designed based on the Preferred Reporting Items of Systematic Reviews and Meta-Analysis (PRISMA) statement guideline [24].

To identify relevant studies conducted on the effects of honey on metabolic parameters in adult patients with T2DM and nondiabetic individuals, the four electronic databases including PubMed/Medline, Web of Science, Scopus, and Cochrane library were searched from 2000 to July 2019 using both MeSH and non-MeSH terms. Notably, the search was restricted to the English language studies.

As possible relevant studies were exported into Endnote Software (version X8), two independent investigators (M.A and N.N) screened all studies based on their titles and abstracts, and those that were found to be potentially relevant were transferred to the next step in which studies were assessed based on their full-text considering eligibility criteria. Besides, to avoid missing any related studies, the reference lists of all eligible studies were hand-searched.

### 2.1. Eligibility Criteria

The PICO framework (P, patients/participants; I, intervention; C, comparison; O, outcome) was used to define the inclusion criteria. Accordingly, studies that met the following criteria were included in the review: (i) study population: subjects with T2DM or nondiabetic individuals aged 18 years and over, (ii) intervention: oral consumption of each type of honey, (iii) comparison: the control group receiving a placebo or not receiving it, (iv) outcome: at least one of the following parameters: anthropometric indices (weight, body mass index (BMI), and waist circumference (WC)), glycemic indices (fasting blood glucose (FBG), hemoglobin A1C (HbA1c), insulin, insulin resistance, and insulin sensitivity), and lipid profile (triglyceride (TG), total cholesterol (TC), low-density lipoprotein cholesterol (LDL-C), and high-density lipoprotein cholesterol (HDL-C)), and (v) duration of intervention equal or longer than 7 days. In addition, only clinical trials with either a parallel or cross-over design were considered eligible in the review.

Human studies with any other design, short-term intervention (less than 7 days), studies on children and adolescents, athletes, other types of diabetes, malignant diseases such as cancer, animal studies, in vitro studies, grey literature (conference papers, theses, and interviews), topical treatments, and studies which examined the effects of honey in combination with other materials were excluded from the study.

### 2.2. Data Extraction

The characteristics of the included studies were extracted by two independent reviewers (M. A and M. J). The data extraction form included the following information: the first author's last name, year of publication, location, study design, gender, mean age, sample size at baseline and the end of the trail, disease background, dosage of honey, type of honey, duration of intervention, other interventions, adjustments, outcomes, and findings.

When there were insufficient data for the included clinical trials, we contacted the corresponding author via e-mail. Unless an answer was received after three times of contact at the end of each week, it was excluded completely or for a specific parameter. Furthermore, when parameters were reported more than twice, only measurements at baseline and the end of the trial were extracted. Apart from honey and control groups, data obtained from the other study groups were not extracted.

### 2.3. Quality Assessment

To assess the quality of the eligible clinical trials, a 5-item Jadad scale was applied [25]. Jadad checklist includes three main items as follows: (i) randomization, (ii) blinding, and (iii) an account of all participants. For the first two items, two scores can be dedicated based on the provided information in the study, and the third one can obtain maximum one score. In general, any clinical trial can obtain a maximum of five scores. In the current systematic review, we considered each study with a minimum of three scores as high quality; otherwise, they were classified into the low-quality group. This section was conducted by two independent reviewers (N. N and M. A).

Any discrepancy in each of procedures was resolved by discussion or consulting with the third reviewer (MH. A) as mentioned earlier.

### 2.4. Data Synthesis

We found high heterogeneity in the included studies. Studies examined various types of honey and had different control groups, study designs, and study subjects. Given that this heterogeneity could not be solved by subgrouping due to limited studies with similar characteristics, we could not pool the studies to conduct a meta-analysis. Therefore, findings were reported only in a qualitative format.

## 3. Results

**Figure 1:**
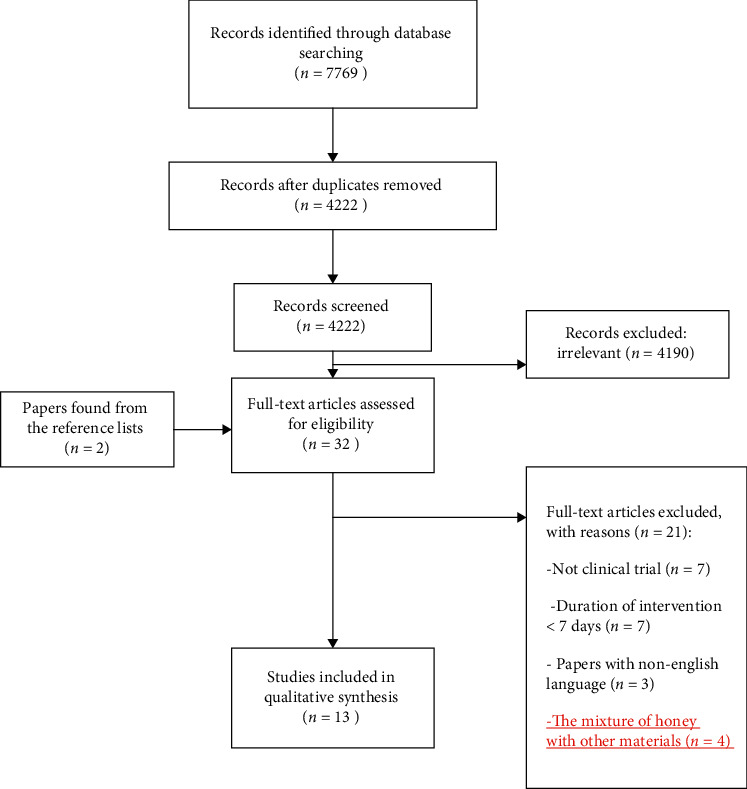
The process to reach the eligible articles.

As depicted in Figure 1, a total of 7769 possible relevant studies (including 3547 duplicates) were identified by searching the electronic databases. After screening titles and abstracts, we found 4190 irrelevant studies excluded from the study. In the next step, full-texts of 32 articles were carefully examined. Two studies were also obtained after checking the reference lists. Twenty-one out of 34 full-text articles were excluded due to the following reasons: not clinical trial (*n* = 7), duration of intervention shorter than seven days (*n* = 7), non-English language studies (*n* = 3), and the mixture of honey with other materials (*n* = 4). In total, 13 studies were eligible and included in the qualitative synthesis.

### 3.1. Study Characteristics

The characteristics of 13 included clinical trials are summarized in Table 1. The clinical trials were published between 2008 and 2019 in Asian countries (*n* = 10), European countries (*n* = 2), and the U.S.A (*n* = 1). All studies except three (cross-over) had a parallel design. All clinical trials were randomized, and 7 studies were single or double-blinded. Sample size ranged from −8 to 128 for both genders (*n* = 8), men (*n* = 2) and women (*n* = 3). They were between 18 years old and 62.8 years old. Different types of honey, including Tualang, Acacia, Rapeseed, Kelulut, and Robinia and six Greek varieties, were studied. Types of honey were not reported in some studies (*n* = 7). The effects of honey was examined in healthy (*n* = 4) and unhealthy individuals (*n* = 9), including patients with T2DM (*n* = 3). The remaining were conducted on patients with glucose intolerance (*n* = 3), high TC (*n* = 1), and overweight/obesity (*n* = 2). The duration of intervention in clinical trials with a parallel design ranged from 8 days to 12 months, and the dosage varied between 5 g/day and 80 g/day.

The included studies examined the impact of oral consumption of honey on glycemic status (*n* = 12), anthropometric indices (*n* = 6), lipid profiles (*n* = 10), and blood pressure (*n* = 3). Based on the Jadad scale, 5 studies had high methodological quality (score ≥3), and the remaining (*n* = 8) had low methodological quality (score <3) (Table 2).

### 3.2. Systematic Review of Studies with Placebo Groups

In 5 clinical trials, the effects of honey on metabolic parameters were compared with placebo or control groups. In a study conducted by Yaghoobi et al., the impacts of natural honey were compared with those of sucrose in both healthy subjects and patients with high risk factors [26]. They found that 70 g/day honey reduced only BMI and FBS with no changes in lipid profile in healthy subjects, while it reduced TG in high risk participants. Changes in other lipid profiles and anthropometric indices were insignificant [26]. Findings of the study by Munstedt et al. revealed that a solution containing 75 g honey only reduced serum levels of LDL-C in women, not men. Comparison of honey with the solution containing 75 g glucose, and fructose showed no changes in TG and TC after 14 days [27]. According to Raatz et al., daily consumption of 50 g/day honey, sucrose, and high-fructose corn syrup (three study groups) increased TG and insulin concentrations in glucose-tolerant and intolerant subjects after 14 days [28]. The mentioned changes were also significant among the study groups. Rasad et al. indicated that compared to sucrose, 80 g/day honey solved in 250 mL water decreased serum levels of FBS with no changes in blood pressure in healthy young subjects after 30 days [29]. Notably, they controlled findings for age, physical activity, and some nutrient intake as confounder factors. Despland et al. compared the effects of consumption of Robinia honey, high free glucose and fructose diet, and low fructose diet (control group) in healthy normal weight men for seven days [30]. All study groups received a weight maintenance diet during the intervention. They found that diet with 25% of total energy from honey or pure fructose-glucose might slightly reduce postprandial blood glucose and insulin, while postprandial TG did not change compared to the control group [30].

Findings were classified into two groups, studies with and without control groups. In the end, the impacts of honey in patients with T2DM were also presented.

### 3.3. The Systematic Review Section for Studies without a Placebo Group (with a Control Group)

A total of seven clinical trials with no placebo groups were included in the study. Majid et al. indicated that natural honey (70 g/days for four weeks) reduced FBS, TG, and LDL-C and increased HDL-C concentrations in healthy young men compared to those continued their usual diet [31]. In their study, Sadeghi et al. demonstrated that 50 g/day honey along with a weight maintenance diet increased HbA1c and reduced waist circumference in patients with T2DM compared to those adhered to only a weight maintenance diet after 8 weeks [32]. However, FBS, insulin, homeostatic model assessment for insulin resistance (HOMA-IR), quantitative insulin sensitivity check index (QUICKI), weight, BMI, hip circumference, and waist-to-hip ratio did not change significantly [32]. Enginyurt et al. compared the effects of three dosages of honey (5, 15, and 25 g/day) with a group receiving no honey. They found that HbA1c was decreased in all intervention groups, but no significant changes were observed in patients with T2DM in terms of lipid profile after 4 months [33]. Mushtaq et al. demonstrated a significant reduction in serum levels of TC, LDL-C, and TG and an increase in HDL-C in groups receiving honey (40 g/day) compared to nonusers of honey in most ethnic groups after 4 weeks. They found that the effects of honey in obese subjects were higher than those in normal weight subjects [23].

Nik Hussein et al. also revealed that 20 g/day Tualang honey along with hormonal replacement therapy (HRT) compared to HRT alone did not have substantial effects on blood pressure, lipid profile, glucose level, BMI, and WC in postmenopausal women after 4 months [34]. Based on the report published by Rashid et al., 30 g/day Malaysian Kelulut honey did not change FBS, lipid profiles, blood pressure, and BMI in patients with IFG after 30 days as compared to control [35]. However, Bahrami et al. demonstrated that natural honey reduced bodyweight, FBS, and LDL-C/HDL-C ratio in patients with T2DM after 8 weeks compared to controls, but no changes were observed in HbA1c and lipid profiles [22]. They examined difference dosages of honey through the intervention, from 1 g/kg/day in the first two weeks to 2.5 g/kg/day in the 7^th^ week and 8^th^ week. Notably, they adjusted findings for baseline values. Rashid et al. showed that consumption of 30 g/day Kelulut honey did not affect the glycemic status, lipid profiles, blood pressure, and BMI compared to those without taking honey after 1 month in patients with impaired fasting glucose [35].

In a clinical trial, the impacts of two kinds of honey (Tualang and honey cocktail) for 12 months and the results showed that honey cocktail increased BMI, while it reduced FBS. In addition, Tualang honey reduced DBP compared to honey cocktail in postmenopausal women [36].

### 3.4. The Effects of Honey in Patients with T2DM

Three clinical trials examined the effects of honey in patients with T2DM [22, 32, 33]. Two clinical trials showed a significant increase in HbA1c following the intake of a minimum 50 g/days honey for 8 weeks [22, 32]. However, Enginyurt et al. showed that following the consumption of 5–25 g/day honey for 4 months, a reduction in HbA1c was observed [33].

## 4. Discussion

The current systematic review showed that oral consumption of honey might have no positive effects on the modulation of metabolic parameters in nondiabetic subjects. In addition, a high intake of honey might increase glucose levels and worsen other metabolic parameters in patients with T2DM. Due to substantial heterogeneity in study design and limited clinical trials, results, however, should be interpreted with great caution.

To the best of our knowledge, this is the first systematic review on the effects of oral consumption of honey on metabolic parameters in diabetic and nondiabetic individuals. Therefore, we cannot compare our findings with an earlier systematic review. We found a mixture of positive, negative, and null effects on metabolic status following the consumption of honey. This discrepancy might be due to differences in mean age, disease background, gender, BMI at baseline, dosage, duration of intervention, other intervention along with taking honey, and ethnic of participants as well as differences in types of honey obtained from various botanical sources.

In most clinical trials included in the current review, the effects of honey were not compared with those of the placebo. This point can cause considerable bias and affect both the internal and external validities of studies. Although this point is more important for subjective outcomes, it can also lead to overestimating or underestimating the real effects of the intervention for objective outcomes [37]. On the other hand, based on the Jadad scale, the most clinical trials had low methodological quality. Due to this issue, we cannot draw a fix conclusion about the effects of honey on each metabolic parameter.

Another weakness of the most included studies was related to reporting findings without controlling confounders, including baseline characteristics, total energy intake, physical activity, and BMI. The main confounder that can affect findings is total energy intake. Honey is a high-nutrient functional food containing 64 kcal in each tablespoon (about 20 g). Therefore, adding honey to diet, particularly in high dosage with no replacement, no changes in daily calorie intake and physical activity can increase body weight and fat mass due to extra calorie intake. Therefore, metabolic parameters can be influenced by these changes. However, only in the two included clinical trials [30, 32], a weight maintenance diet was recommended along with honey consumption, and in most studies, findings were not reported after adjusting for such confounding factors. The importance of this issue was clarified in the study of Despland et al. They found that a diet containing 25% of total energy intake after designing individualized weight maintenance diets did not show harmful effects on glucose and TG levels, and even, it showed reduction effects [30].

Accordingly, it seems that the negative effects of honey on metabolic parameters reported in some included clinical trials might be related to the added calorie and fructose content of honey. Some studies demonstrated that hypertriglyceridemia was associated with fructose or glucose administered in a hypercaloric diet, while no significant effects were found in a weight maintenance diet [38, 39]. Some previous studies have also showed that fructose can increase TG concentrations and stimulate hepatic de novo lipogenesis [40, 41].

Notably, the amount of fructose and fructose/glucose ratio in honey can contribute to different findings. Based on meta-analyses, postprandial TG can increase following the consumption of fructose greater than 50–60 g/day [42], and an increase in FBS is observed with daily dosage exceeding 100 g [42, 43]. However, the amount of fructose from honey and whole diet was reported in only two studies [28, 30]. The average daily intake of fructose was 95 g in the study of Despland et al. [30]. The fructose content of honey in another clinical trial was also 40 g/100 g of honey [28]. Fructose to glucose ratio in honey is another possible factor affecting metabolic parameters, and it differs among various types of honey. Among the included clinical trials, only Despland et al. reported the ratio, and it was 1.7 [30]. This ratio may affect both the GI of honey that is important, particularly for patients with DM and glycemic status.

It is possible that some factors, including polyphenols and other antioxidant ingredients of honey, can blunt the harmful effects of fructose on metabolic parameters. Such components are influenced by botanical sources and geographical locations [12]. Therefore, identifying the types of honey with therapeutic effects can be helpful. However, the different types of honey and their nutritional value were provided only in limited studies. Thus, we were not able to report findings based on classifications by the mentioned factors. It seems that producing honey with identified and controlled amounts of components along with adding some nutrients or materials, including probiotics and prebiotics in some cases, can be helpful, particularly for unhealthy subjects.

There were only three studies conducted on patients with T2DM [22, 32, 33]. Although this number is insufficient to conclude how much honey can be allowed for diabetic patients, we can conclude that it is not necessary to delete honey completely from diabetic diets and only restriction and to replace with other carbohydrate sources along with the maintenance of total energy intake is sufficient. One out of three studies conducted on diabetes examined different dosages of honey (5–25 g/days) [33]. The results showed that these dosages not only had no negative effect on metabolic parameters but also reduced HbA1C. However, higher dosage (50 g/day) increased HbA1c in patients with T2DM [32].

It is not fully understood by which mechanisms honey can affect metabolic profiles. However, some potential pathways are suggested. The main possible mechanisms are related to the ingredients of honey, particularly polyphones and flavonoids with anti-inflammatory and antioxidant properties. Through the suppression of inflammatory pathways, reducing free radicals, and helping to rebalance oxidant and antioxidant factors, modulation of glycemic status, lipid profiles, and other metabolic factors can be occur. Other mechanisms are reduction in body weight, increased satiety, delay in gastric emptying, and modulation of appetite hormones including neuropeptide Y and ghrelin due to phenolic components and oligosaccharides content [32], affecting C-peptide, stimulating beta cells of the pancreas due to antioxidant components [35], an increase in uptake of hepatic glucose and glycogen synthesis and storage due to fructose [33].

However, this study has some limitations that should be addressed. They are as follows: (i) due to high heterogeneity in the methodology of the included clinical trials and limited studies with similar characteristics, we were not able to do a meta-analysis, (ii) studies conducted on other metabolic profiles such as inflammatory and antioxidant parameters were not included, (iii) we could not determine cutoff points for dosage of honey with no negative effect on metabolic parameters due to high heterogeneity. However, doing a systematic review on this topic for the first time, examining the quality of studies, providing results separately for both diabetic and nondiabetic subjects were the strengths of this systematic review.

## 5. Conclusion

The current systematic review revealed that oral consumption of honey might have no beneficial effects on the modulation of metabolic status in nondiabetic subjects. Even high intake of honey might increase glucose levels and worsen other metabolic parameters in patients with T2DM. Due to substantial heterogeneity in study design, low quality in most clinical trials, and limited included studies, results, however, should be interpreted with great caution. More high quality randomized controlled clinical trials on different types of honey (with determined physicochemical properties) with various dosages and longer duration of the intervention are necessary to clarify the effects of honey in diabetic and nondiabetic individuals.

## Figures and Tables

**Table 1:** The characteristics of the included studies in the current systematic review.

Author/Year	Location	Study design	Gender (male/female)	Mean age	Sample size at baseline	Disease background	Sample size at end	Dosage of honey	Duration	Botanical source of honey/type	Other intervention	Adjustment	Findings	
Sadeghi et al./2019	Iran	RCT (cross-over)	Both	57.5	Total (n = 53) Honey (n = 27) Control (n = 26)	T2DM	Total (n = 42) Honey (n = 18) Control (n = 24)	50 g/day	8 weeks (4 weeks wash out)	Milk vetch	Weight maintenance diet for the control and honey group	—	HbA1c	↔
													WC Waist-to-height ratio	↓
													Glucose Weight BMI Insulin level HC Waist-to-hip ratio HOMA-IR HOMA-*β* QUICKI	↔

Rashid et al./2019	Malaysia	RCT	Both	51.6	Total (*n* = 60) Kelulut honey (*n* = 30) Control (*n* = 30)	IFG	Total (*n* = 64) Kelulut honey (*n* = 27) Control (*n* = 27)	30 g	30 days	—	—	—	FBS TC HDL-C LDL-C BMI SBP DBP	↔

AB Wahab et al./2018	Malaysia	RCT (double-blind, parallel)	F (postmenopause)	58	Total (*n* = 100) Tualang honey (*n* = 50) Honey cocktail (*n* = 50)	T2DM (*n* = 12) HTN (*n* = 49) HLP (*n* = 51)	Total (*n* = 98) Tualang honey (*n* = 49) Honey cocktail (*n* = 49)	20 g/day	12 months	Honey cocktail (contains honey-bee bread-royal jelly)	—	—	FBS BMI DBP	↑ ↓ ↓

Enginyurt O. et al./2017	Turkey	RCT	Both	18–80	Total (*n* = 64) Intervention/patient (*n* = 32) Control/healthy (*n* = 32)	T2DM (using metformin)	Total (*n* = 64) Intervention/patient (*n* = 32) Control/healthy (*n* = 32)	5, 15, and 25 g/d	4 months	—	—	—	HbA1c (all of dose) TC (doses of 5 and 25 g/d)	↓
													TG HDL	↔
													LDL	↔
														↓
														↔

Despland et al./2017	Switzerland	RCT (cross-over)	M	—	Total (*n* = 8)	Healthy	Total (*n* = 8)	25% total energy	8 days	—	—	—	Weight Height BMI Fat mass Lean mass SBP DBP Resting heart rate Total TG	↔
													Chylomicrons-TG LDL Glucose Insulin	↓
Raatz et al./2015	North Dakota	RCT (cross-over)	Both	GT: 38.9 IGT: 52.1	Total (*n* = 89) GT (*n* = 52) IGT (*n* = 37)	GT-IGT	Total (*n* = 55) GT (*n* = 28) IGT (*n* = 27)	50 g carbohydrate from honey, HFCS, and sucrose	2 weeks (2–4 weeks wash out)	—	—	—	Weight BMI Glucose Insulin HOMA_IR-glucose iAUC Insulin iAUC SBP DBP TC HDL-C LDL-C TG	↔

Majid et al./2014	Pakistan	RCT	M	Honey: 20 Control: 20	Total (*n* = 70) Honey (*n* = 35) Control (*n* = 35)	Healthy	Total (*n* = 63) Honey = 32 Control = 31	70 g	4 weeks	—	—	—	FBS TG	↔
													TC LDL-C	↓
													HDL-C	↑

Rasad et al./2014	Iran	RCT (double-blind)	M	Honey: 21.53 Sucrose: 24.23	Total (*n* = 60) Honey (*n* = 30) Sucrose (*n* = 30)	Healthy	Total (*n* = 60) Honey (*n* = 30) Sucrose (*n* = 30)	80 g honey in 250 ml water	6 weeks	—	—	Age, physical activity, and some nutrient intake	FBS	↓
													DBP SBP	↔

Nik Hazlina et al./2012	Malaysia	RCT	F	55.4	Total (*n* = 82) Tualang honey (*n* = 41) HRT (*n* = 41)	Healthy postmenopausal	Total (*n* = 79) Tualang honey (*n* = 40) HRT (*n* = 39)	20 g/day	4 months	—	—	Baseline values, age, BMI, WC, and duration of menopause	BMI WC SBP DBP TC TG HDL FBS	↔
Mushtaq et al./2011	Pakistan	Randomized controlled clinical trial	Both	Normal weight, honey: M, 46.25 and F, 42.8 Normal weight, no honey: M, 42.5 and F, 42.9 Obese, honey: M, 41.27 and F, 40.06 Obese, no honey: M, 39.87 and F, 39.66	Total (*n* = 160) Honey: obese (*n* = 40) and normal weight (*n* = 40) No honey: obese (*n* = 40) and normal weight (*n* = 40)	Obese/normal weight	Total (*n* = 128) Honey: obese (*n* = 32) and normal weight (*n* = 32) No honey: obese (*n* = 32) and normal weight (*n* = 32)	40 g/day	4 weeks	—	—	—	BMI: obese and normal weight, LDL-C and normal weight, HDL Normal weight: female	↔
													TC, normal weight (male Baloch and Pathan and female Baloch) TC, obese (male Baloch and Punjabi and female Baloch, Punjabi, and Hzara) TG, normal weight (male Baloch and female Punjabi) TG, obese (male all ethnic groups and female Baloch, Pathan, and Punjabi) LDL-C, obese (male all ethnic groups and female Baloch and Pathan)	↓
													HDL-C, normal weight (male Pathan and Punjabi) HDL-C, obese (male Baloch, Pathan and Hzara, and female Baloch and Pathan)	↑

Munstedt et al./2009	Germany	RCT	Both	Honey: 62.6 Honey-comparable sugar solution: 58.8	Total (*n* = 60) Honey group (*n* = 30) Honey-comparable sugar solution group (*n* = 30)	High cholesterol	Total = 60 Honey group: 30 Honey-comparable sugar solution group: 30	75 g	14 days	Mixed blossom honey from Europe, central America, and South America	—	—	Women: LDL	↔
													General: TG, LDL, HDL, and TG	↔
Bahrami et al./2009	Iran	RCT	Both	57.2	Total (*n* = 54) Honey (*n* = 28) Control (*n* = 26)	T2DM	Total (*n* = 48) Honey (*n* = 25) Control (*n* = 23)	First 2 weeks: 1 g/kg/d Second 2 weeks: 1.5 g/kg/d Third 2 weeks: 2 g/kg/d Last 2 weeks: 2.5 g/kg/d	8 weeks	Natural unprocessed honey	—	Baseline values	Bodyweight TG TC LDL-C FBS LDL/HDL	↓
													HDL-C HbA1c	↑

Yaghoobi et al./2008	Iran	RCT	Both	41.2	Total (*n* = 60) Honey: 40 Control: 20	Obese or overweight	Total (*n* = 55) Honey: 38 Control: 17	70 g/250 ml tap water	30 days	Natural unprocessed honey	—	—	Normal variable: in subjects with normal values: BMI and FBG	↓
													Patients with TG >150 mg/dl	
													Bodyweight Body fat TC LDL-C HDL-C	↔

RCT, randomized controlled clinical trial; T2DM, type 2 diabetes mellitus; WC, waist circumference; BMI, body mass index; HC, hip circumference; HOMA, homeostasis model assessment insulin resistance; QUICKI, quantitative insulin sensitivity check index; FBS, fasting blood sugar; DBP, diastolic blood pressure; HTN, hypertension; HLP, hyperlipidemia; TC, total cholesterol; TG, triglycerides; HDL-C, high-density lipoprotein cholesterol; LDL-C, low-density lipoprotein cholesterol; VLDL, very low-density lipoprotein; iAUC, incremental area under curve; GI, glycemic index; SBP, systolic blood pressure; FBS, fasting blood sugar; GT, glucose tolerance; ↔, no significant effect; ↓, decrease; ↑, increase.

**Table 2:** The Jadad score for the included clinical trials (*n* = 13).

Author (year)	Randomization score	Blinding score	Account of patients score	Total score
Sadeghi et al. (2019)	2	1	1	4
Rashid et al. (2019)	0	0	0	0
AbWahab et al. (2018)	2	2	1	5
Enginyurt et al. (2017)	1	0	0	1
Despland et al. (2017)	1	0	1	2
Raatz et al. (2015)	1	0	1	2
Majid et al. (2014)	2	1	1	4
Rasad et al. (2014)	1	1	1	3
Nik Hussein et al. (2012)	1	0	1	2
Mushtaq et al. (2011)	0	0	0	0
Mu¨nstedt et al. (2009)	1	1	1	3
Bahrami et al. (2009)	1	0	1	2
Yaghoobi et al. (2008)	1	0	0	1

## Data Availability

No data were used to support this study.
